# Facile Synthesis of FAPbI_3_ Nanorods

**DOI:** 10.3390/nano10010072

**Published:** 2019-12-29

**Authors:** He Huang, Linzhong Wu, Yiou Wang, Alexander F. Richter, Markus Döblinger, Jochen Feldmann

**Affiliations:** 1 Chair for Photonics and Optoelectronics, Nano-Institute Munich, Department of Physics, Ludwig-Maximilians-Universität München (LMU), Königinstr 10, 80539 Munich, Germany; linzhong.wu@physik.uni-muenchen.de (L.W.); Yiou.Wang@physik.uni-muenchen.de (Y.W.); alexander.richter@physik.uni-muenchen.de (A.F.R.); 2 Department of Chemistry, Ludwig-Maximilians-Universität München, Butenandtstrasse 5–13 (E), 81377 Munich, Germany; m.doeblinger@lmu.de

**Keywords:** perovskite, FAPbX_3_, nanorods, ligand-assisted re-precipitation

## Abstract

Metal halide perovskites are promising materials for a range of applications. The synthesis of light-emitting perovskite nanorods has become popular recently. Thus far, the facile synthesis of perovskite nanorods remains elusive. In this work, we have developed a facile synthesis to fabricate FAPbI_3_ nanorods for the first time, demonstrating a high photoluminescence quantum yield of 35–42%. The fabrication of the nanorods has been made possible by carefully tuning the concentration of formamidine-oleate as well as the amount of oleic acid with pre-dissolved PbI_2_ in toluene with oleic acid/oleylamine.

## 1. Introduction

Metal halide perovskites, with the general formula of ABX_3_ (where A and B represent monovalent and divalent cations, respectively, and X is a monovalent halide anion e.g., Cl^−^, Br^−^ or I^−^ or their mixture), are promising materials for a range of optical applications such as solar cells, light-emitting diode devices (LEDs), photodetectors, and so on [1,2,3,4]. Following the first colloidal synthesis of the organic-inorganic CH_3_NH_3_PbBr_3_ perovskite nanocrystals (NCs) in 2014 by Galian, Perez-Prieto and co-workers [1]. Zhong and co-workers introduced a ligand-assisted re-precipitation (LARP) technique to produce CH_3_NH_3_PbX_3_ (X = Cl, Br, I) NCs [5]. At nearly the same time, Kovalenko and co-workers developed all-inorganic CsPbX_3_ perovskite NCs [2]. These perovskite NCs, different from bulk perovskite, have the potential to be used as emitters for the next generation of optoelectronic devices on account of their outstanding performance including near-unity photoluminescence (PL) quantum yield (QY), precisely tunable wavelengths of emission, solution processability, and defect tolerance [4,6,7,8]. Different shapes of perovskite NCs have also been successfully fabricated including nanoplatelets, nanowires, nanobelts, and nanorods [3,9,10]. Among them, the synthesis of light-emitting perovskite semiconductor nanorods (NRs) has recently become popular to generate linearly polarized light, which is essential for the next generation of liquid crystal displays. The application of NRs will improve the efficiency in the utilization of backlight power [11]. Until now, perovskite NRs can be fabricated by indirect synthetic methods such as chemical transformation from Cs_4_PbBr_6_ nanopolyhedrons [12] or chemical cutting of nanowires [13]. The direct synthetic methods reported so far normally require higher energy ultrasonication [14] or high temperatures [15]. Because of the ionic nature of perovskite compounds, the controllability of both morphology and size remains unsatisfactory in direct synthesis. Thus far, a facile approach to synthesize perovskite NRs straightforwardly remains elusive.

In this work, we have developed a novel synthetic route to fabricate FAPbI_3_ NRs for the first time, which demonstrates a high PL QY of 35–42%. Different wavelengths of emission (on FAPbBr_3_ and FAPbBr*_x_*I_3−*x*_ NRs with similar dimensions) could be controlled via carefully tailored processes of anion-exchange. The fabrication of the NRs has been made possible by the fine-tuning of the concentration of formamidine-oleate and the amount of oleic acid with pre-dissolved PbI_2_ in toluene with oleic acid/oleylamine. Furthermore, a possible pathway for the formation of NRs was proposed to understand the underlying mechanism.

## 2. Materials and Methods

Materials: Formamidine acetate (99%), lead (II) iodide (PbI_2_, 98.5%), oleic acid (OA, technical grade 90%) were purchased from Alfa Aesar (Karlsruhe, Germany), oleylamine (OLA, 80–90%), toluene (99.85%) were purchased from Acros Organics (Geel, Belgium). OA and OLA were dried for 1 h for further use. All the other chemicals were kept in the glove box and used as received.

Methods: Formamidine-oleate (FA-oleate) precursor was prepared by dissolving 1 mmol formamidine acetate in 10 mL of oleic acid under ultrasonication.

PbI_2_ precursor solution was prepared by dissolving 0.1 mmol PbI_2_ powder in a mixture of 100 µL of oleic acid, 100 µL oleylamine and 10 mL toluene (concentration: 0.01 mol/L) at 80 °C under continuous stirring.

FAPbI_3_ NRs: 60 µL of FA-oleate was added into 2 mL of PbI_2_ precursor solution under vigorous stirring at room temperature. After 1 min of stirring, the solution was immediately centrifuged at 14,500 rpm for 5 min and the precipitate was redispersed in 1 mL of toluene. The final product as the supernatant was obtained by further centrifugation at 2000 rpm for 2 min. We estimate the synthetic yield is lower than 1%.

Characterization: The absorption and photoluminescence spectra were obtained with a Cary 60 UV-Vis spectrophotometer and a Varian Cary Eclipse fluorescence spectrophotometer (Agilent Technologies, Santa Clara, CA, United States), respectively. The photoluminescence quantum yields (PLQYs) were determined by an absolute method using an integrating sphere with its inner face coated with BENFLEC^®^ (Edinburgh Instruments, Livingston, United Kingdom) coupled to a Horiba Fluorolog 3 spectrofluorometer (Horiba, Kyoto, Japan). The morphology of the NRs was characterized by transmission electron microscopy (TEM) operating at an accelerating voltage of 80–100 kV (JEOL JEM-1011). High-angle annular dark-field scanning transmission electron microscopy (HAADF-STEM) images were acquired using an FEI Titan Themis microscope (FEI, Hillsboro, OR, United States) operating at 300 kV. A probe semiconvergence angle of ~17 mrad was used. X-ray diffraction (XRD) measurements were performed by a Philips X-Pert Xray diffractometer (Philips, Amsterdam, Netherlands) using Cu Kα radiation (λ = 1.5418 Å).

## 3. Results and Discussion

**Figure 1:**
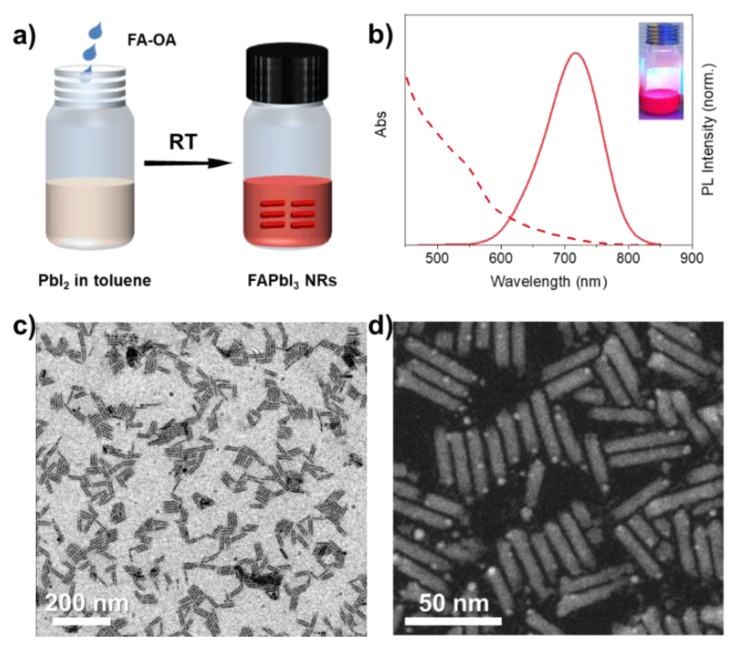
(**a**) Schematic illustration of the synthesis of perovskite nanorods (NRs) by addition of FA–oleate complex into the PbI_2_–ligand solution under ambient conditions. (**b**) Ultraviolet–visible (UV/Vis) absorption (dashed line) and photoluminescence (PL) spectra (solid line) of perovskite NRs. The inserted photograph shows the colloidal solution under UV illumination. (**c**) Transmission electron microscopy (TEM) image and (**d**) High-angle annular dark-field scanning transmission electron microscopy (HAADF-STEM) image of FAPbI_3_ perovskite NRs.

Figure 1a shows the synthetic process of FAPbI_3_ NRs. The method used here is adapted from our previous publication [16]. In a typical synthesis, 60 µL of formamidine-oleate (0.1 M in OA) was added into 2 mL of PbI_2_ precursor (0.01 M in toluene with OA and OLA) solution under vigorous stirring at room temperature. After 1 min of stirring, the solution was immediately centrifuged and re-dispersed in toluene for further characterizations. It is worth mentioning that the quality of chemicals is critical for the successful synthesis of perovskite NRs. Absorbing a tiny amount of water by formamidine acetate and toluene could possibly lead to the decomposition of the final product. An impurity of OA or OLA would also result in a low yield of the product, making it difficult to isolate the NRs.

To understand the optical properties of the as-prepared FAPbI_3_ NRs, the colloidal solutions containing these NRs were characterized by UV/Vis absorption and PL spectroscopy (Figure 1b). The PL peak is centered at 717 nm, which agrees with an absorption onset at 679 nm, exhibiting a small Stokes shift (38 nm). The PLQY of the FAPbI_3_ perovskite NRs is 42%. We did the stablity measuremnt of NRs prepared by this approach under UV illumination (365 nm, 12 W power) at ambient conditions (see Figure S2). The morphology of the FAPbI_3_ NRs has been characterized by transmission electron microscopy (TEM) and STEM. Figure 1c shows that the perovskite NRs are quite monodisperse and tend to self-assemble on the TEM grid to form small islands, which is typical as observed in TEM images of perovskites. The average length and width of the NRs determined from STEM are ≈ 29 and 6 nm, respectively. The XRD spectra of FAPbI_3_ NRs shows a cubic perovskite crystal structure (α-phase) with some contributions of δ-phase (Figure S1). It is unlikely to be α/δ-phase junction in the FAPbI_3_ NRs [17].

**Figure 2:**
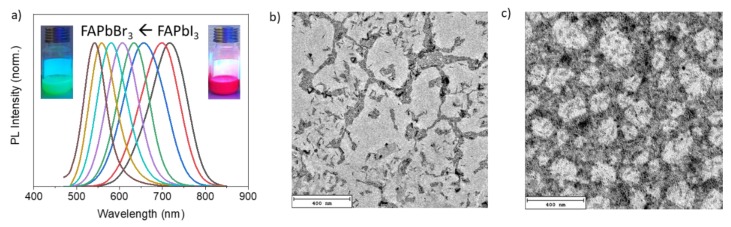
PL spectra of FAPbX_3_ (X = Br, and I) NRs after halide-exchange reactions performed on the pristine FAPbI_3_ NRs (**a**) insert: photographs of the colloidal solutions under UV illumination. Representative TEM images of FAPbBr*_x_*I_3−*x*_ (**b**) and FAPbBr_3_ (**c**).

Although FAPbI_3_ NRs were directly synthesized, the same method was not as successful of for the fabrication of FAPbCl_3_ and FAPbBr_3_ NRs because of the lower quality and synthetic yield. Further optimization could be favored for their direct synthesis in order to obtain NRs with both higher quality and synthetic yield. Alternatively, we applied a well-known halide ion-exchange approach to acquire NR dispersions with various bandgaps and wavelengths of emission by tuning their halide composition, starting from directly synthesized FAPbI_3_ NRs [18,19]. By the halide exchange reaction, the emission of the resultant FAPbX_3_ NRs varied from red to green as the halide proportions gradually changed from I^−^ to Br^−^ (Figure 2a). By increasing the amount of bromide precursor, the wavelength of emission shifts from 717 to 541 nm. This indicates that the Br^−^ ions have replaced most of the I^−^ ions in the pristine FAPbI_3_ NRs. The absorption and PL for FAPbI_3_ and FAPbBr*_x_*I_3−*x*_ perovskite nanorods are shown in Figure S3. As shown in Figure 2b and c, the halide ion-exchange reaction preserved the rod shape of FAPbI_3_ NRs with similar lengths and widths. For FAPbBr*_x_*I_3−*x*_ NRs (PL peak at 633 nm), it still maintained the shape of most of the NRs. When the ion-change proceeded further, FAPbBr_3_ NRs shows some dilation because of some reconstruction alongside with the exchange itself. Apart from NRs, quasi-nanocubes gradually appeared because of the breakdown of NRs (Figure 2c). The PL QY of ion-exchanged FAPbBr_3_ is 35%, slightly lower than the direct-synthesized FAPbI_3_ (42%). We found that it was difficult to employ ion-exchanged FAPbBr_3_ to obtain FAPbCl_3_ NRs because of the stability issues. Most of the NRs will break into quasi-nanocubes with only a few remaining.

**Figure 3:**
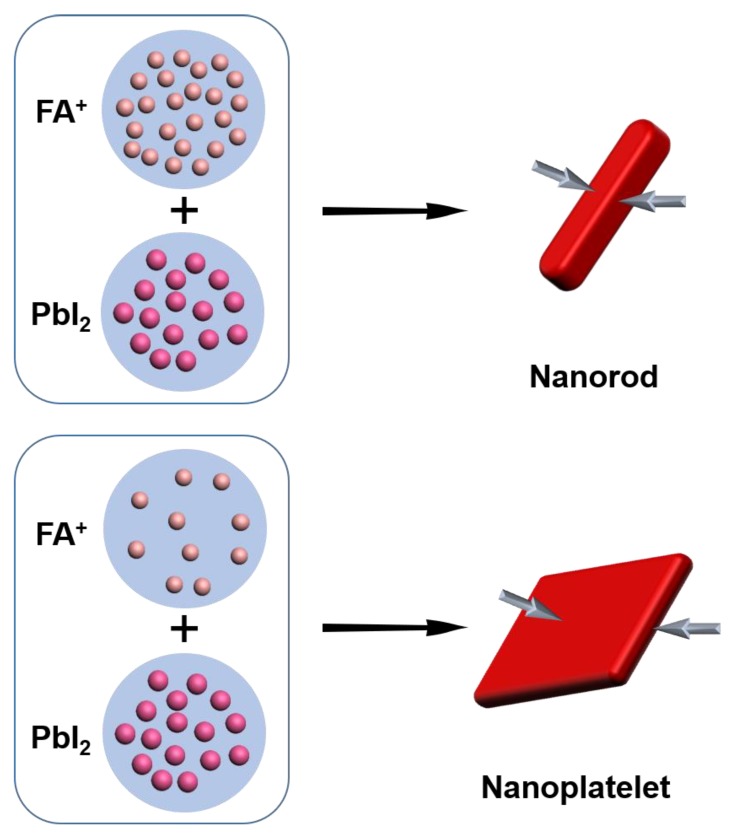
Schematic illustration showing the transformation of precursor-ligand complexes into either FAPbX_3_ nanorods or nanoplatelets depending on FA concentration because of different surface to volume ratios.

As schematically depicted in Figure 3, the morphology of NCs changed from 2D nanoplatelets to 1D nanorods as the concentration of FA-oleate increased. Similar to our previous work, [16] nanoplatelets were obtained at a lower concentration of FA. This suggests that anisotropic growth is favorable at a higher FA concentration when the ratio of FA/Pb remains low. In general, a reduction in the concentration of reactants slows down the rate of reaction, and consequently the growth rate of nanocrystals. It might be that the formation of nanorods or nanoplatelets is determined by the ratios between surface and volume. A higher concentration of FA leads to more confinement of perovskite growth in both directions, hence forming the structure of NRs.

## 4. Conclusions

In summary, we have presented a facile synthetic strategy to fabricate FAPbI_3_ NRs for the first time. Different wavelengths of emission on FAPbBr_3_ and FAPbBr*_x_*I_3−*x*_ NRs with similar dimensions have also been realized by an anion-exchange method. The fabrication of the NRs has been made possible by fine-tuning the concentration of FA and the amount of OA. At present, this approach has not yet been able to produce highly stable perovskite NRs, but further optimization must be applied to enhance the robustness of such NRs. This work opens a new era in the facile synthesis of metal halide perovskite nanorods with diverse potential applications in optoelectronic devices such as LEDs with polarization.

## Supplementary Materials

The following are available online at https://www.mdpi.com/2079-4991/10/1/72/s1, Figure S1: XRD data of FAPbI_3_ perovskite nanorods. Figure S2: The stability of FAPbI_3_ perovskite nanorods under UV illumination (365 nm, 12W) at ambient conditions: Change in the relative PL intensity of perovskite NC solutions vs UV illumination time. Figure S3: The representative absorption and PL for FAPbI_3_ and FAPbBr_x_I_3-x_ perovskite nanorods.
