# Supplementary material for: Facile Synthesis of FAPbI3 Nanorods
Source: Nanomaterials (Basel). 2019 Dec 29;10(1):72. doi: 10.3390/nano10010072 (PMC7022980; doi:10.3390/nano10010072)
Supplement: Supplementary file 1 [file nanomaterials-10-00072-s001.pdf]

Supplementary Information for

Facile Synthesis of $FAPbI_3$ Nanorods

He Huang[1,*], Linzhong Wu[1], Yiou Wang[1], Alexander F. Richter[1], Markus Döblinger[2], Jochen Feldmann[1,*]

1 Chair for Photonics and Optoelectronics, Nano-Institute Munich, Department of Physics, Ludwig-Maximilians-Universität München (LMU), Königinstr. 10, 80539, Munich, Germany;
Nanosystems Initiative Munich (NIM) and Center for NanoScience (CeNS), Schellingstr. 4, 80799, Munich, Germany

2 Department of Chemistry, Ludwig-Maximilians-Universität München, Butenandtstrasse 5-13 (E), 81377 Munich, Germany

* Correspondence: he.huang@ physik.uni-muenchen.de; feldmann@lmu.de

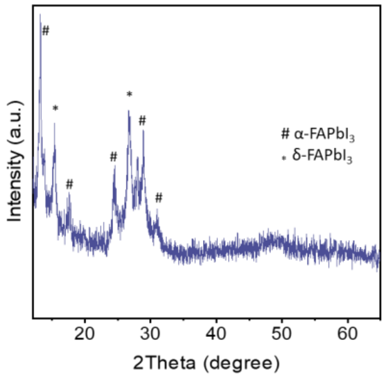

Figure S1: XRD data of $FAPbI_3$ perovskite nanorods.

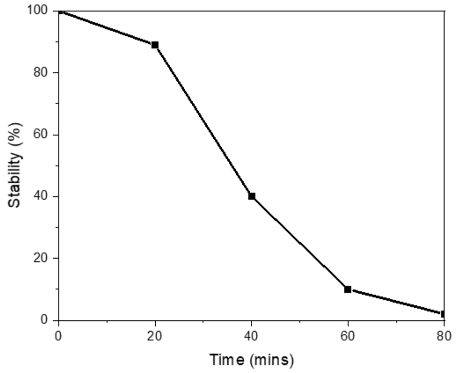

Figure S2: The stability of $FAPbI_3$ perovskite nanorods under UV illumination (365 nm, 12W) at ambient conditions: Change in the relative PL intensity of perovskite NC solutions vs UV illumination time.

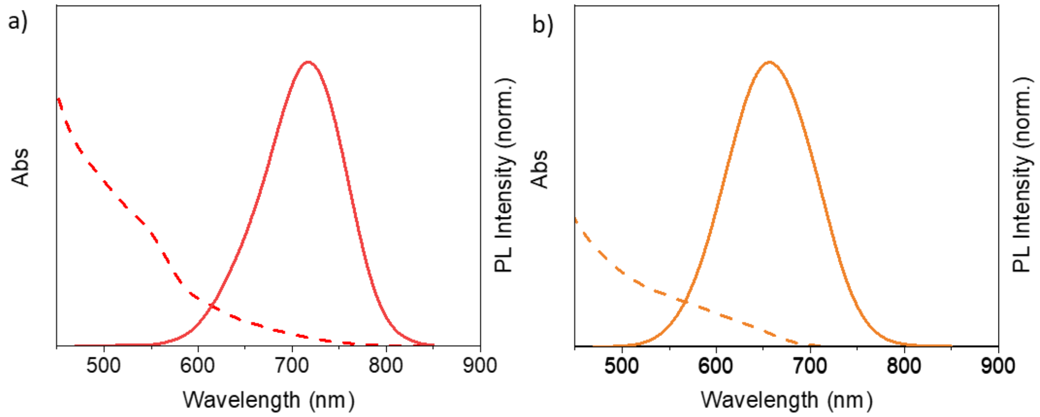

Figure S3: The representative absorption and PL for $FAPbI_3$ and $FAPbBr_xI_{3-x}$ perovskite nanorods.
